# Redox Imbalance Is Associated with Neuronal Apoptosis in the Cortex of Neonates Gestated Under Chronic Hypoxia

**DOI:** 10.3390/antiox14060736

**Published:** 2025-06-15

**Authors:** Esteban G. Figueroa, Rodrigo L. Castillo, Adolfo A. Paz, Matías Monsalves-Alvarez, Francisca Salas-Pérez, Ximena Calle, Tamara A. Jiménez, Emilio A. Herrera, Alejandro Gonzaléz-Candia

**Affiliations:** 1 Laboratory of Fetal Neuroprogramming, Institute of Health Sciences, Universidad de O’Higgins, Rancagua 3655000, Chile; esteban.figueroa@uoh.cl; 2 Institute of Health Sciences, Universidad de O’Higgins, Rancagua 3655000, Chile; francisca.salas@uoh.cl (F.S.-P.); ximena.calle@uoh.cl (X.C.); 3 Department of Internal Medicine East, Faculty of Medicine, Universidad de Chile, Santiago 7500922, Chile; rodrigouch@uchile.cl; 4 Critical Patient Unit, Hospital del Salvador, Santiago 7500922, Chile; 5 Laboratory of Vascular Function & Reactivity, Pathophysiology Program, ICBM, Faculty of Medicine, Universidad de Chile, Santiago 8330015, Chile; adolfo.paz@ug.uchile.cl (A.A.P.); tamara.jimenez.espinosa@edu.udla.cl (T.A.J.); eaherrera@uchile.cl (E.A.H.); 6 Exercise and Rehabilitation Sciences Institute, Faculty of Rehabilitation Sciences, Universidad Andrés Bello, Santiago 7610196, Chile; matias.monsalves@unab.cl; 7 Geroscience Center for Brain Health and Metabolism (GERO), Santiago 7800003, Chile

**Keywords:** oxidative stress, intrauterine chronic hypoxia, mitochondrial dysfunction, neuron death

## Abstract

Gestational chronic hypoxia impacts prenatal development, leading to fetal growth restriction (FGR), defined as the fetus’s failure to reach its genetic growth potential. Postnatal hypoxia in the cerebral tissue can induce a redox imbalance and mitochondrial dysfunction, consequently increasing neuronal death. However, these data cannot necessarily be extrapolated to prenatal hypoxia. In this regard, this study aims to describe the effect of gestational hypoxia on redox balance and apoptosis cell death mechanisms in the prefrontal cortex of guinea pigs. Ten Guinea pig (*Cavia porcellus*) pregnant dams were utilized in this study; five gestated in normoxia (Nx; three newborn males, and two females) and five gestated under chronic hypobaric hypoxia (Hx; two newborn males, and three females). We monitored the pregnancies by ultrasound examinations from gestational days 20 to 65 (term ~ 70). At birth, pups were euthanized, and the fetal brain was collected for cellular redox measurement, mitochondrial enzyme expression, and apoptosis assay. Gestation under hypoxia induced an imbalance in the expression of anti- and pro-oxidant enzymes, resulting in increased oxidative stress. Additionally, a decrease in cytochrome I and III expression and neuronal density in the neonatal prefrontal cortex was observed. Finally, DNA fragmentation was increased by the TUNEL assay in the brain tissue of newborns gestated under chronic hypoxia. Our findings demonstrate the association of gestational hypoxia with oxidative stress and neuronal death in newborns, which may predispose to neuronal dysfunction in adulthood.

## 1. Introduction

The intrauterine environment in which the fetus develops conditions the physiology and determines its health in adult life [1,2]. Several internal and external factors can affect the intrauterine environment, such as maternal nutrition, stress, infections, and gestational hypoxia [2,3]. During normal gestational development, the placenta is the organ in charge of supplying oxygen and nutrients to the fetus through the umbilical fetal interconnection. However, a decrease in the mother’s blood oxygen supply to the fetus can cause prenatal hypoxia and FGR [4]. FGR is defined as a condition where the fetus does not reach its growth potential due to decreased blood flow through the placenta [4]. The physiological response to this gestational hypoxia by the fetus is the development of the phenotype called the “brain-sparing effect”, in which blood flow is redistributed to maintain vital organs, such as the brain and heart, at the expense of normal peripheral blood supply and growth [5]. However, although brain sparing is an adaptation to hypoxia, the programming by oxidative stress and its impact on the brain health of the newborn is poorly understood.

Free radical generation is present in the placenta and fetus from the beginning of pregnancy, contributing to normal fetal development. During gestation, the antioxidant activity protects the developing embryo from oxygen-free radical damage. The increase in oxidative stress is a determining factor for brain development, as demonstrated by Neonatal Hypoxic–Ischemic Encephalopathy (HIE) models. Oxidative stress is an imbalance in homeostasis between the production of reactive oxygen species (ROS) by cellular pro-oxidant sources and antioxidant systems; this can be an alteration of either a decrease in antioxidants or an increase in pro-oxidants or both, resulting in damage in different organs [6]. However, organs with greater sensitivity to oxygen, such as the cardiovascular and brain systems, are more likely to generate oxidative stress and cellular damage.

The cellular pro-oxidant sources induced by hypoxia are NADPH oxidase (NOX) and mitochondrial uncoupling, which are responsible for ROS production [7,8]. The NOX family, specifically NOX2 and NOX4, contributes significantly to the production of ROS during hypoxia and in the brain tissue. NOXs are biochemically different in their regulation, expression, and activity but retain their ability to use NADPH as an electron donor to reduce molecular oxygen to the superoxide anion radical (•O_2_-) [9,10,11]. On the other hand, under postnatal hypoxia, the mitochondrial electron transport chain uncouples, which generates a leak of electrons, specifically in complexes I and III, accumulating •O_2_- in the inner mitochondria membrane, deriving in oxidative stress [12]. In addition, postnatal hypoxia can also decrease antioxidant capacity, further increasing oxidative stress [13]. The mechanisms that control ROS metabolization include enzymatic and non-enzymatic pathways. The enzymatic antioxidant machinery consists of superoxide dismutase (SOD), catalase (CAT), and glutathione peroxidase (GPX), which contribute to the neutralization of •O_2_- and the subsequent reduction of hydrogen peroxide (H_2_O_2_) to water [13]. On the other hand, non-enzymatic mechanisms include antioxidant molecules such as vitamins E and C in addition to β-carotenes, ubiquinone, lipoic acid, and urate, and its action is based on direct scavenging [14].

The neonatal brain is susceptible to oxidative stress for different reasons, including the following: (i) the rapid increase in tissue oxygen concentration associated with the extrauterine environment, considering the hypoxic gestational environment [15,16]; (ii) their antioxidant machinery is still immature, and an elevated fraction of iron free of transferrin in their system increases susceptibility to oxidative damage [17]; and (iii) the vulnerability of the brain to oxidative stress is accentuated by its high oxygen consumption and (iv) the presence of unsaturated fatty acids, which makes it prone to lipid peroxidation [18]. These oxidative stress mechanisms have been associated with brain development and function, in addition to causing cell death, inflammation, and brain deterioration, contributing to the development of postnatal neurodegenerative diseases [19]. Considering the above, this study aims to describe the effect of chronic hypoxia on brain redox balance and cell death mechanisms, affecting neuronal density in the neonatal cortex of guinea pigs gestated under hypoxia.

## 2. Materials and Methods

### 2.1. Animals and Experimental Groups

This study used 10 adult female Pirbright White Guinea Pigs (*Cavia porcellus*). Animals were housed under standard conditions (35–40% humidity, 20–21 °C, and 12:12 h light–dark cycle) and had a specialized diet for this species (LabDiet 5025, Guinea Pigs, 25–30 g/day). At estrous, the female was paired with a male for one day in normoxic conditions, and 20 days later, the pregnancy was confirmed by visualization of the gestational sac by ultrasound examination. Only one neonate per litter was used for this protocol; hence, the number of mothers corresponded to the number of neonates used in our study. However, the ultrasound determinations were performed in all fetuses of each litter due to the impossibility of separating these results at the prenatal level. At birth, 5 neonates were assigned to the normoxic group (Nx, 3 newborn males, and 2 females), and 5 neonates were assigned to the hypobaric hypoxic group (Hx, 2 newborn males, and 3 females). On gestational day (GD) 30, all animals from both groups were introduced to a hypobaric chamber in conditions of normoxia (Nx, controls, 720 torrs) or Hypoxia (Hx, 470 torrs) until delivery. At birth, the newborn underwent euthanasia with an overdose of sodium thiopentone (100 mg/kg, IP), subsequently the brain tissue was quickly obtained from molecular biology, histology, and immunohistochemistry. A coronal cut of 1 cm of thickness of the prefrontal cortex was fixed in 4% *v*/*v* paraformaldehyde in PBS for 24 h at 4 °C. Subsequently, the tissue was embedded in paraffin and cut into 10 μm thick slides for histology and immunohistochemistry assays as previously described [20].

### 2.2. Pre- and Postnatal Biometry Assessment

A portable ultrasonograph (Z6 VET, Mindray, Shenzhen, China) with an L14-6P linear ultrasound transducer performed biometrical prenatal assessments. Ultrasonographic examinations were conducted in conscious and non-sedated animals, gently restrained by an expert operator. Biparietal diameter (BPD), abdominal circumference (AC), and cranial circumference (CC) were measured by ultrasound examination during pregnancy and averaged at GD30–GD35, GD40–GD45, and GD60–GD65. Prenatal flow velocity patterns were quantified through the pulsatility index (PI) in the umbilical artery (UA) and middle cerebral artery (MCA) by Doppler ultrasound during GD60–GD65. The cerebroplacental ratio was calculated as MCA PI to UA PI [21]. Weight, biparietal diameter (BD), and cerebral weight were determined as previously described at birth [21].

### 2.3. Protein Expression and Activity Assay in Total Brain

Protein expression for SOD1, SOD2, SOD3, CAT, GPX1/2, NOX2, NOX4, COX2, Total OXPHOS, NF-κB, TNFα, iNOS, IL-1β, IL-8, IL-10, Bcl-xL, BAX, cleaved-Caspase 3, Tom20 and α/β tubulin were determined in total cerebral lysates. Briefly, the membranes were incubated with primary antibodies (anti-SOD1, Santa Cruz Biotechnology (Dallas, TX, USA), sc-101523; anti-SOD2, Santa Cruz Biotechnology, sc-133134; anti-SOD3, Santa Cruz Biotechnology, sc-271170; anti-CAT, Abcam Laboratories (Cambridge, UK), ab1877; anti-GPX1/2, Santa Cruz Biotechnology, sc-133160; anti-NOX2, Santa Cruz Biotechnology, sc-130543; anti-NOX4, Santa Cruz Biotechnology, sc-518092; anti-COX2, Abcam Laboratories, ab102005; Total OXPHOS Rodent WB Antibody Cocktail, Abcam Laboratories, ab110413; anti-NF-κB, Abcam Laboratories, ab16502; anti-TNFα, Santa Cruz Biotechnology, sc-12744; anti-iNOS, Santa Cruz Biotechnology, sc-7271; anti-IL-1β, Santa Cruz Biotechnology, sc-12742; anti-IL-8, Santa Cruz Biotechnology, sc-376750; anti-IL-10, Santa Cruz Biotechnology, sc-365858; anti-Bcl-xL, Cell Signaling (Danvers, MA, USA), #2764; anti-BAX, Cell Signaling, #2772; anti-Tom20, Santa Cruz Biotechnology, sc-17764; and anti-α/β-tubulin, Cell Signaling, #2148, respectively). The signals were revealed using chemiluminescent substrates (SuperSignal West Pico, #34080 and Femto, #34095, Thermo Scientific, Waltham, MA, USA), digitalized by a scanner (ChemiDoc™ Imaging Systems, Bio-Rad, Hercules, CA, USA), quantified by densitometry, and normalized by α/β-tubulin or Tom20 signal using Scion Image software version 4.02, as described elsewhere [22]. On the other hand, the antioxidant enzyme activities in cerebral homogenate and plasma were measured using the Superoxide Dismutase (SOD) Activity Assay Kit (706002, Cayman Chemical Company, Ann Arbor, MI, USA), Catalase Activity Assay Kit (707002, Cayman Chemical Company, Ann Arbor, MI, USA), Glutathione Peroxidase Assay Kit (703102, Cayman Chemical Company, Ann Arbor, MI, USA), Glutathione reduced (GSH), an oxidized (GSSG) ratio Detection Assay Kit (Colorimetric) (ab239709, Cayman Chemical Company, Ann Arbor, MI, USA) and 8-Isoprostane ELISA Kit (516351, Cayman Chemical Company, Ann Arbor, MI, USA), according to the manufacturers’ guidelines. As previously described [23], total protein concentration was used for normalization.

### 2.4. Immunolocalization of Proteins in the Cerebral Cortex

4-Hydroxynonenal (4-HNE), 3-Nitrotyrosine (NT), and cleaved caspase-3 were assessed in coronal sections of the prefrontal cortex (anti-4-HNE, Abcam Laboratories, ab46545; anti-NT, Santa Cruz Biotechnology, sc-32757; and anti-cleaved caspase-3, Cell Signaling, #9664). Briefly, the tissue sections were exposed to retrieval buffer 1X for antigen retrieval (Target Retrieval Solution, Dako, Carpinteria, CA, USA) at 120 °C for 25 min. The primary antibodies were incubated in bovine serum albumin 1% (1:100) for three hours. Then, the slides were incubated for one hour with a Mouse/RabbitPolydetector DAB HRP Brown System (Bio SB^®^, Goleta, CA, USA). Finally, diaminobenzidine revealed the immunoreaction and the nuclear stain was performed with Harris hematoxylin. All slides were digitally acquired at 400X (Olympus BX-41, Tokyo, Japan) and analyzed as specific reddish-brown pixel count per area relative to the positive control. The neuron was selected for mark quantification, and pixels were quantified using Adobe Photoshop (CS5 extended version 12.0, San Jose, CA, USA). The mark intensity (pixels) was then divided by the area of each arterial layer (pixels/m^2^), as previously described [20].

### 2.5. Apoptosis Detection in the Cerebral Cortex

Prefrontal cortex slice sections 10 μm in thickness were used to measure neuronal death using the TUNEL Dead End™ Fluorometric TUNEL System apoptosis detection kit (Promega, Madison, WI, USA). This kit measures fragmented DNA from apoptotic cells by catalytically incorporating fluorescein-12-dUTP into the 3′-OH ends of DNA using the recombinant terminal deoxynucleotidyl transferase (rTdT) enzyme. rTdT forms a polymeric tail utilizing the principle of the TUNEL (TdT-mediated dUTP NickEnd Labeling) assay. Fluorescein-12-Dut-labeled DNA was then directly visualized by fluorescence microscopy (Zeiss microscope, Jena, Germany). DAPI was utilized to detect nuclear localization [24].

### 2.6. Statistical Analyses

Statistical analyses were performed using Graph Pad Prism 10.0 (San Diego, CA, USA). First, the normality of the data was checked through both Shapiro–Wilk and Kolmogorov–Smirnov tests. In addition, possible outlier values were detected through the Grubbs test, which were excluded from the subsequent comparisons. The results are expressed as mean ± SEM. Multiple unpaired *t*-tests were used to compare the prenatal biometry, and all other results were compared with a nonparametric Student *t*-test. to assess the association of the apoptosis markers with the neuronal density, these were correlated with a Pearson test. Statistical significance was considered when *p* < 0.05. Grubb’s test (using 5% significance level critical values) detected outlier ratios [22,23].

## 3. Results

### 3.1. Pre- and Postnatal Biometric Variables

Fetal BPD, AC, CC, and placental biometry were measured using ultrasound in three stages of the pregnancy: GD 30–35, 40–45, and 60–65. Fetuses gestated in chronic hypoxia showed a decrease in their BPD during GD 30–35 and 40–45; in addition, AC decreased towards the end of gestation from GD 40–45 onwards in fetuses gestated in chronic hypoxia compared to control fetuses, as shown in Table 1. Finally, in fetuses gestated under hypoxia, only a significant decrease in CC was observed between days GD 30–35 compared to the normoxic group, as described in Table 1. On the other hand, hypoxic fetuses at GD 60–65 showed an increase in the CPR and a decrease in the placental biometry (such as length, thickness, and area) in chronic hypoxic fetuses compared to normoxic fetuses, as shown in Table 1 and Supplementary Figure S1. Gestational hypoxia resulted in decreased birth weight and a higher BPD/weight ratio in the hypoxic group compared to the normoxic group. However, cerebral weight was similar between the groups, as shown in Table 1.

### 3.2. Antioxidant Capacity of the Postnatal Brain

**Figure 1:**
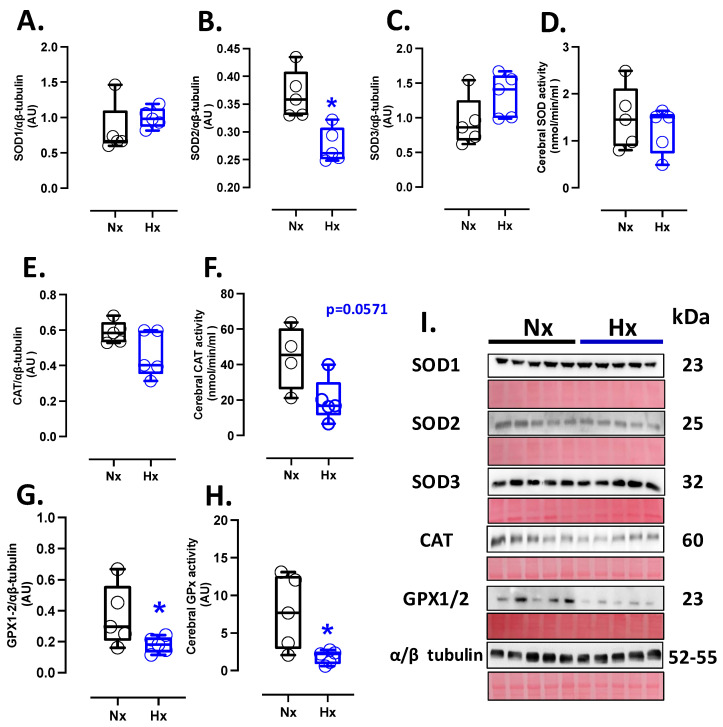
Antioxidant protein expression and activity in the neonatal brain. Protein expression by Westen blot and activity levels by ELISA kit of (**A**) SOD1, (**B**) SOD2, (**C**) SOD3, (**D**) total SOD activity, (**E**) CAT, (**F**) CAT activity, (**G**) GPX1-2, and (**H**) GPX total activity. (**I**) shows the blots with the molecular weight and ponceau corresponding to each membrane for each protein. Groups are neonates gestated in normoxia (Nx, *n* = 5, black bars, and circles) or hypobaric hypoxia (Hx, *n* = 5, blue bars, and circles). Data expressed as mean ± S.E.M. and compared using a Mann–Whitney *t*-test. Significant differences (*p* ≤ 0.05): * vs. Hx.

The protein levels of SOD1 and SOD3 showed similar expressions among the groups analyzed (Figure 1A,C), while SOD2 protein expression decreased in the hypoxic group compared to the normoxic one (Figure 1B). Nevertheless, no changes in SOD activity were observed between groups (Figure 1D). Concerning protein levels and CAT activity in neonatal brain tissue, our data showed no differences between the analyzed groups (Figure 1E,F). Similarly, the protein levels and activity of the GPX1-2 showed no significant difference between groups, as shown in Figure 1G,H.

### 3.3. Pro-Oxidant Protein Levels and Oxidative Stress Marker in the Postnatal Brain

**Figure 2:**
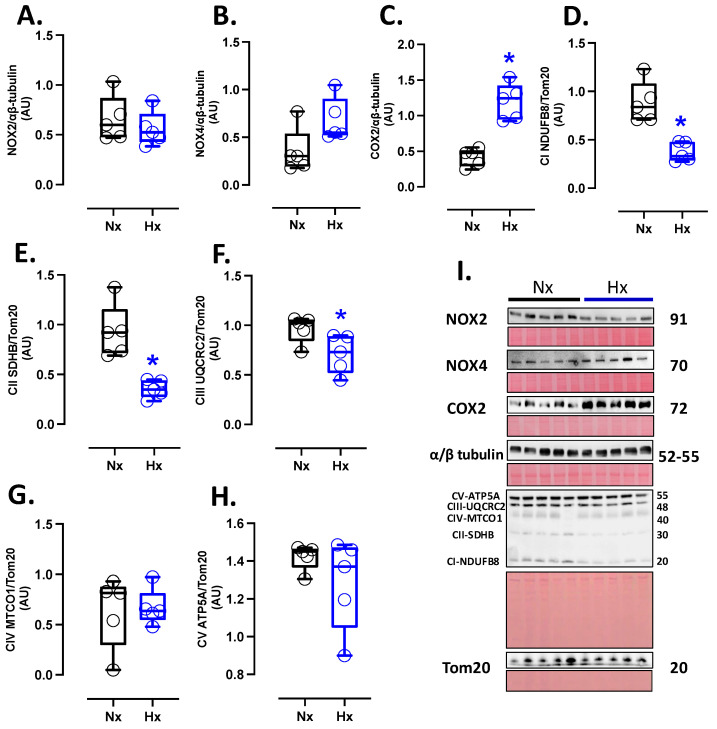
Pro-oxidants protein expression in the neonatal brain. Protein expression by Westen blot of (**A**) NOX2, (**B**) NOX4, (**C**) COX2, and OXPHOS: (**D**) Cytochrome I or NDUFB8 (NADH:ubiquinone oxidoreductase subunit B8), (**E**) Cytochrome II or SDHB (Succinate dehydrogenase cytochrome b), (**F**) Cytochrome III or UQCRC2 (ubiquinol-cytochrome c reductase core protein 2), (**G**) Cytochrome IV or MTCO1 (Mitochondrially Encoded Cytochrome C Oxidase I), and (**H**) Cytochrome V or ATP5A (ATP Synthase Subunit Alpha, Mitochondrial). (**I**) shows the blots with the molecular weight and ponceau corresponding to each membrane for each protein. Groups are neonates gestated in normoxia (Nx, *n* = 5, black bars, and circles) or hypobaric hypoxia (Hx, *n* = 5, blue bars, and circles). Data expressed as mean ± S.E.M. and compared using a Mann–Whitney *t*-test. Significant differences (*p* ≤ 0.05): * vs. Hx.

**Figure 3:**
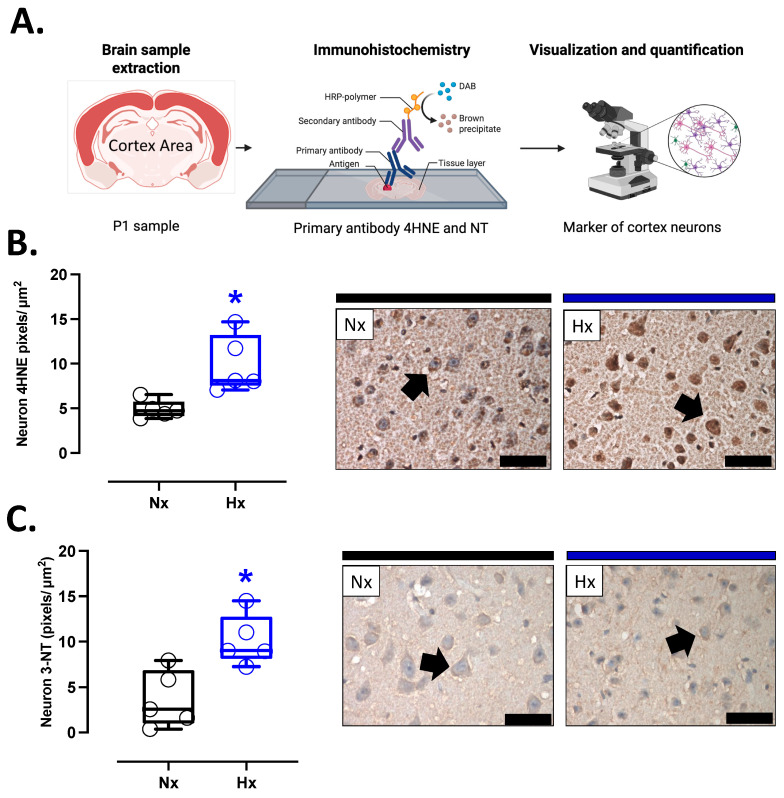
Oxidative stress markers in the neonatal cortex. (**A**) schematic representation of the experimental procedure, (**B**) Immunohistochemistry of 4HNE (4-Hydroxynonenal), and (**C**) NT (3-Nitrotyrosine) with representative micrographs (40×, panel below). Groups are neonates gestated in normoxia (Nx, *n* = 5, black bars and circles) or hypobaric hypoxia (Hx, *n* = 5, blue bars and circles). Bar in the micrographs = 100 μm. Reddish-brown color indicates positive staining and the arrows show cortical neurons. Data are expressed as mean ± S.E.M. and compared using a Mann–Whitney *t*-test. Significant differences (*p* ≤ 0.05): * vs. Hx.

Pro-oxidant sources were measured in total brain homogenate. The isoforms NOX2 and NOX4 showed no differences between groups (Figure 2A,B). However, the protein levels of COX2 were increased in the Hx group relative to the Nx group (Figure 2C). Quantification of mitochondrial cytochrome proteins showed a decrease in CI, CII, and CIII levels of proteins for the hypoxia group compared to the normoxia group (Figure 2D–F). However, no significant changes were observed in CIV and CV (Figure 2G,H). On the other hand, the oxidative stress markers, 4HNE and NT, were increased in the cortex in the hypoxic group relative to the normoxic group (Figure 3).

### 3.4. Inflammatory Protein Levels in the Postnatal Brain

**Figure 4:**
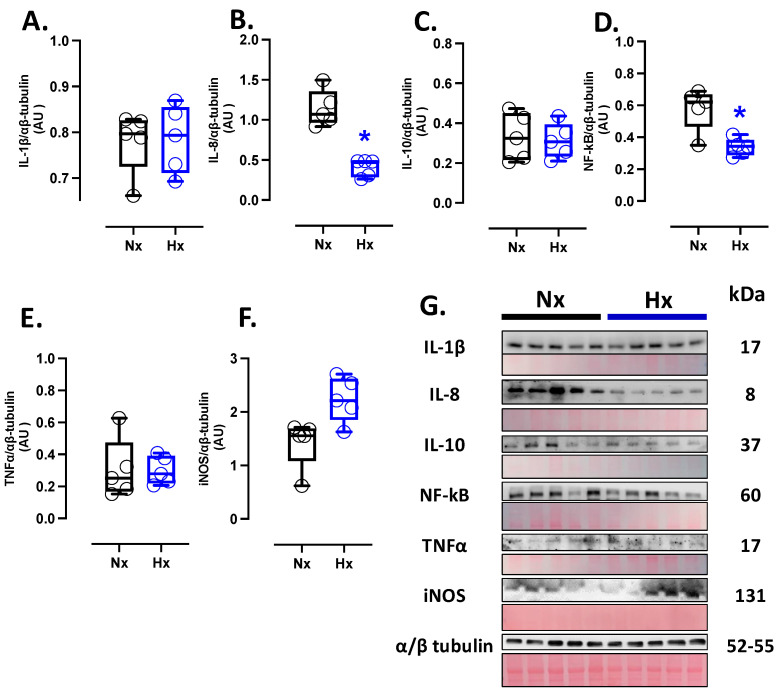
Inflammation-related protein expression in the neonatal brain. Protein expression by Westen blot of (**A**) IL-1β (Interleukin 1β), (**B**) IL-8 (Interleukin 8), (**C**) IL-10 (Interleukin 10), (**D**) NF-κB (Nuclear factor kappa-light-chain-enhancer of activated B cells), (**E**) TNFα (tumor necrosis factor-alpha), and (**F**) iNOS (Inducible nitric oxide synthase). (**G**) shows the blots with the molecular weight and ponceau corresponding to each membrane for each protein. Groups are neonates gestated in normoxia (Nx, *n* = 5, black bars, and circles) or hypobaric hypoxia (Hx, *n* = 5, blue bars and circles). Data are expressed as mean ± S.E.M. and compared using a Mann–Whitney *t*-test. Significant differences (*p* ≤ 0.05): * vs. Hx.

Inflammatory markers were measured in total brain homogenate. The protein levels of IL-8 and NF-κB were decreased in the hypoxic group compared to the normoxic group (Figure 4B,D); however, no changes were observed in the protein levels of IL-1β, IL-10, TNFα, and iNOS (Figure 4A,C,E,F). Finally, the expression of iNOS in the total brain was similar between the analyzed groups (Figure 4F).

### 3.5. Apoptosis and Neuronal Density in Postnatal Brain Cortex

**Figure 5:**
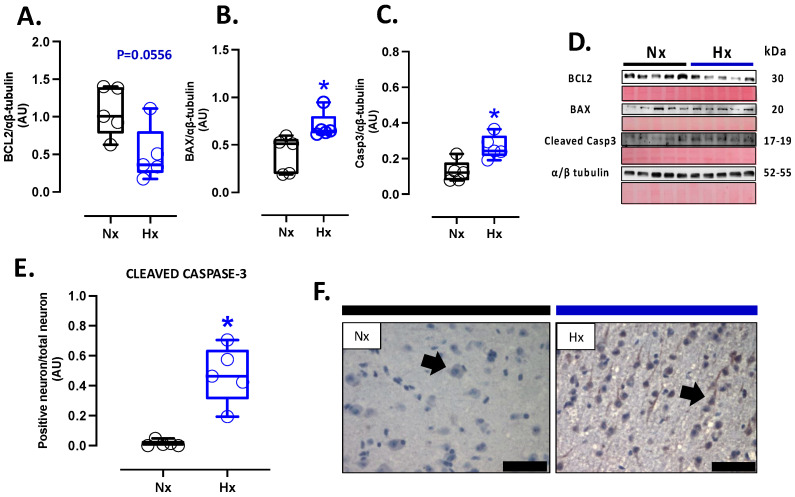
Apoptotic-related protein expression in the neonatal cortex. Protein expression by Westen blot of (**A**) BLC-2, (**B**) BAX, (**C**) Cleaved Casp-3 (cleaved-caspase 3), (**D**) representative image of the western blots with the molecular weight and ponceau corresponding to each membrane for each protein, (**E**) immunohistochemistry of Cleaved Casp-3 and (**F**) Cleaved Casp-3 with representative micrographs (40×, panel below). Groups are neonates gestated in normoxia (Nx, *n* = 5, black bars, and circles) or hypobaric hypoxia (Hx, *n* = 5, blue bars and circles). Bar in the micrographs = 100 μm. Reddish-brown color indicates positive staining, and the arrows show cortical neurons by immunohistochemistry. Data expressed as mean ± S.E.M. and compared using a Mann–Whitney *t*-test. Significant differences (*p* ≤ 0.05): * vs. Hx.

**Figure 6:**
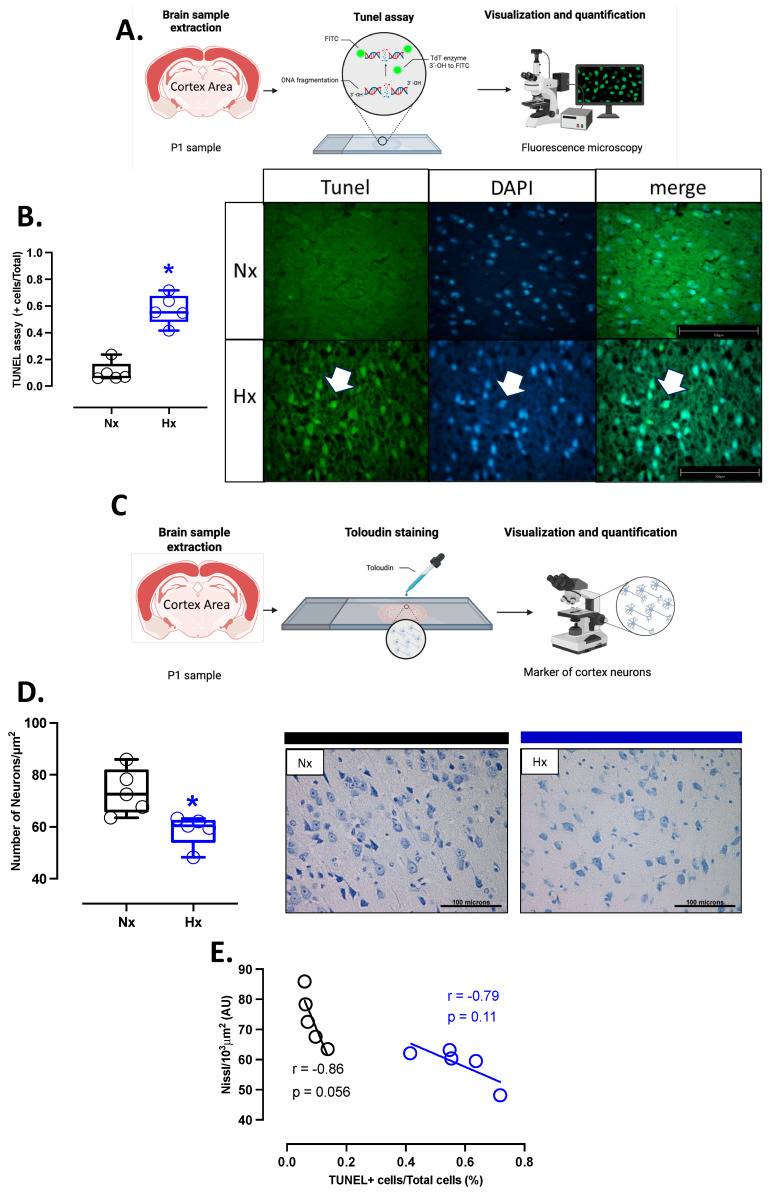
Apoptosis in the neonatal cortex. (**A**) schematic representation of the experimental TUNEL fluorometric assay used to determine neuron apoptosis, (**B**) quantification of TUNEL-positive neurons in the prefrontal cortex with representative micrographs (40×, panel below), (**C**) schematic representation of the experimental toloudin assay in neurons of the prefrontal cortex, (**D**) neuron cell density in the prefrontal cortex by toloudin staining, and (**E**) correlation between neuronal TUNEL staining and neuron density in the prefrontal cortex. Groups are neonates gestated in normoxia (Nx, *n* = 5, black bars, and circles) or hypobaric hypoxia (Hx, *n* = 5, blue bars and circles). Bar in the micrographs = 100 μm. Data expressed as mean ± S.E.M. and compared using a Mann–Whitney *t*-test. Significant differences (*p* ≤ 0.05): * vs. Hx.

Apoptosis markers were evaluated in the total brain tissue. The protein levels of BCL2 were decreased in the hypoxic group relative to the normoxic one (Figure 5A). In contrast, BAX and cleaved Caspase 3 protein levels increased in the hypoxic group compared to the normoxic group (Figure 5B,C). In addition, the immunolocalization of the cleaved Caspase 3 protein in neurons showed a significant increase in the Hx group compared to the Nx group (Figure 5D). On the other hand, the DNA fragmentation measurement through the TUNEL assay showed an increased positive signal in cortex neurons in the hypoxia group compared to the normoxia group (Figure 6A,B), in addition, this study observed a positive correlation between TUNEL+ neurons and nitrotyrosine mark in cortical neurons, as shown in the figure (Figure S2). Finally, neuronal density quantified by toloudin staining (Figure 6C) showed a decrease in the number of neurons of the neonates gestated under hypoxia compared to the normoxia group (Figure 6D).

## 4. Discussion

This study demonstrates that alterations in the oxidative stress balance are associated with detrimental brain effects due to exposure to chronic hypoxia during pregnancy. The adverse environmental conditions to which an individual is exposed during pregnancy, such as intrauterine hypoxia, can predispose them to an increased risk of diseases during adulthood with limited treatment options [25,26]. Under physiological conditions, ROS are mediators that fulfill specific functions that contribute to cellular homeostasis [27]. This is achieved by maintaining a balance between the production of oxidative stress-generating sources and the antioxidant machinery that protects from cellular damage; however, during the fetal period, it has been demonstrated that the antioxidant machinery is still immature, reaching maturity at term and in the first days after birth [28,29]. Hypobaric hypoxia during gestation induces FGR associated with decreased prenatal and postnatal biometry. The latter involves the fetal redistribution of cardiac output related to the vasodilation of the MCA and an increased cerebroplacental ratio, which suggests a brain-sparing phenotype as a chronic response mechanism to hypoxia [5]. This *Guinea pig* model for FGR has characteristics that are very different from conventional murine models. First, the mother’s womb environment is similar to that of humans; second, gestational evolution timing is mostly like humans and has a relatively long gestation compared to classic murine models; third, using the rate of brain growth through gestation and birth as an indicator of development, guinea pigs can be categorized as prenatal brain developers because of their similarities with neurodevelopment in humans [30]. Although the mechanistic pathways linking gestational hypoxia, oxidative stress, and placental dysfunction have not been fully elucidated, in sheep gestated and born in chronic hypoxia, there is an increased oxidative stress associated with the programming of pro-oxidant sources and a decrease in the cardiopulmonary antioxidant machinery [23]. Although our data do not show results in the activity or expression of the antioxidant systems of CAT and SOD in brain tissue, we observed a decrease in the protein levels of GPx1 and a reduction in the total activity in brain tissue of the GPXs. This enzymatic system is susceptible to the cytosolic redox state since it depends on Glutathione (GSH), which is vulnerable to oxidation (GSSG) by uncoupling [31].

There are several ROS sources in the brain. The Nox family proteins are involved in various signaling pathways, including brain adaptation to different physiological and pathophysiological stresses. The main isoforms expressed in the brain parenchyma are Nox2 and Nox4 [10]. While Nox2 and Nox4 seem to be regulated by hyperoxia and hypoxia in postnatal models [32], their contribution to oxidative stress remains to be elucidated in models of prenatal hypoxia. In the present study, we did not observe changes in total brain tissue’s Nox2 and Nox4 levels. NOX can be induced by transactivation through proinflammatory molecules [33]; according to our data, gestational hypoxia in brain tissue decreased the levels of IL-8 without changing IL1β and TNFα. Moreover, the decrease in NF-κB (p65 subunit) blunted the NOX activation by inflammation, although postnatal and intermittent hypoxia induces pathways associated with HIF-dependent inflammation according to the previous antecedents [34]. However, one limitation of our study is that we analyzed whole brain homogenate and did not assess NOX levels in different areas or at a cellular level in the brain parenchyma.

COX-2 is expressed under normal conditions in the CNS and contributes to fundamental brain functions; however, COX-2 can be induced by post-translational modifications associated with oxidative stress and proinflammatory responses [35]. Our data showed increased levels of lipoperoxidation (4HNE) and protein nitration (nitrotyrosine) due to oxidative stress in the neonatal cortex. On the other hand, assuming that the brain is the most metabolically active tissue, the primary source of ATP is produced in the mitochondria. Under hypoxic conditions, oxygen availability decreases, leading to a reduction in ATP production. The decrease in ATP results in the accumulation of radicals in the electron transport chain, as observed in HIE models [36]. Our results indicate that the main complexes involved in radical generation (complex I, II, and III) decrease, favoring mitochondrial dysfunction. An increase in oxidative stress and mitochondrial dysfunction has been documented in uteroplacental dysfunction, which underlies the pathogenesis of preeclampsia and FGR [12].

On the other hand, studies in animal models of FGR have reported an increase in activated microglia and astrogliosis, indicative of inflammatory responses [37,38]. Neuroinflammation involves an increase in proinflammatory cytokines (IL-1β, IL-8, and TNFα) and a decrease in anti-inflammatory cytokines (IL-10) [39,40]. Proinflammatory cytokines are critical in postnatal acute hypoxia-ischemia brain injury, affecting neonatal brain development [41,42]. Both IL-1β and IL-8 are glycoproteins involved in cellular communication and are secreted in response to injuries [43]. The release of IL-8 and IL-1β in cerebrospinal fluid after a brain injury has been associated with blood–brain barrier (BBB) dysfunction, facilitating the entry of systemic proinflammatory cytokines into the fetal brain [44,45,46]. Our results indicate that chronic hypoxia exposure during gestation decreases inflammatory markers, specifically IL-8 and NF-κB. A possible explanation is attributed to the timing of hypoxia exposure during gestation used for the FGR model and the immaturity of the inflammatory system in response to perinatal stress [47]. FGR caused by intrauterine hypoxia significantly decreased neurotrophic markers such as nerve growth factor (NGF), neurotrophin-3 (NT-3), and neurotrophin-4 (NT-4) in the FGR group compared with the fetus at the appropriate gestational age [48,49]. In addition, in guinea pig models exposed to hypoxemia for 14 days (GD 46–49) and studied at 64 days postnatal, an increase in proinflammatory cytokines was observed, leading to the loss of neuronal cells [38]. Another study using uterine artery ligation for inducing FGR from gestational day 30 did not show changes in the number of neurons but did affect neuroglial development [37,50]. Although this study does not focus on neurodevelopment associated with gestational hypoxia, sufficient data support our proposal related to the immaturity of the resident proinflammatory system of the brain parenchyma [51].

Several studies have demonstrated that ROS and oxidative stress are pivotal in apoptosis [52,53]. Our results indicate an increased expression of BAX (proapoptotic molecule) and cleaved caspase 3 (apoptosis effector), which could be involved in the neuronal decrease observed in the neonatal cortex. Caspase-3 is the most abundant caspase in the brain and appears to play a crucial role during normal development and brain injuries. In addition, the redox regulation of caspase activity seems to involve post-translational modifications of their catalytic site cysteine residue. The catalytic site cysteines of most caspases are susceptible to oxidation, as demonstrated by previous studies, in which cellular exposure to hydrogen peroxide induces the activation of effector caspases [54]. On the other hand, oxidative stress causes damage to the mitochondrial membrane, allowing the release of cytochrome C (via the intrinsic pathway), which binds to apoptosis protease activating factor 1 (APAF1), subsequently activating caspase-9 and leading to caspase-3 activation [55]. All these data indicate that the presence of mitochondrial dysfunction and oxidative stress caused by gestational hypoxia in the brains of guinea pigs can activate the intrinsic pathway of apoptosis and create an imbalance in the pro- and antiapoptotic molecules [56,57], generating the permeabilization of proapoptotic agents from the mitochondria to the cytosol triggering neuronal apoptosis [58]. However, further studies are needed to determine the effect of neuronal apoptosis on neuronal capacity and the clinical impact of gestational hypoxia.

## 5. Conclusions

Gestational hypoxia induces a brain-sparing phenotype in guinea pig neonates. Additionally, we demonstrated an association between decreased antioxidant sources and increased pro-oxidant sources in the prefrontal cortex, resulting in brain oxidative damage. Furthermore, gestational hypoxia has the potential to lead to mitochondrial dysfunction due to decreased cytochrome I and III expression, accounting for reduced ATP production. These findings ultimately associate them with gestational hypoxia-induced neuronal death through an imbalance between proapoptotic and antiapoptotic factors. Our results establish the groundwork that gestational hypoxia may elicit neuropathologies, as demonstrated in other hypoxia models.

## Figures and Tables

**Table 1:** Fetal and neonatal biometry.

**Prenatal Biometry and Doppler Across Pregnancy**						
	**Nx**	**Hx**	**Nx**	**Hx**	**Nx**	**Hx**
	30–35 GD		40–45 GD		60–65 GD	
Biparietal Diameter (mm)	8.82 ± 0.26	7.13 ± 0.47 *	13.66 ± 0.32	11.48 ± 0.60 *	18.89 ± 0.22	17.65 ± 0.41
Abdominal circumference (mm)	3.45 ± 0.15	2.64 ± 0.19	6.02 ± 0.43	4.39 ± 0.26 *	8.78 ± 0.35	7.21 ± 0.30 *
Cranial Circumference (mm)	3.16 ± 0.09	2.80 ± 0.09 *	4.52 ± 0.10	3.92 ± 0.07	5.79 ± 0.09	5.31 ± 0.09
Cerebral medial artery (PI)	NA	NA	NA	NA	1.09 ± 0.06	1.55 ± 0.28
Umbilical arteria (PI)	NA	NA	NA	NA	1.07 ± 0.06	0.97 ± 0.07
Cerebro-placental ratio (AU)	NA	NA	NA	NA	1.04 ± 0.10	1.63 ± 0.24 *
**Postnatal Biometry (at Birth)**						
	**Nx**	**Hx**				
Birth weight (g)	114.20 ± 14.14	80.48 ± 4.51 *				
Biparietal Diameter/weight (AU)	0.26 ± 0.02	0.20 ± 0.01 *				
Cerebral weight (g)	2.05 ± 0.04	1.93 ± 0.10				
Cerebral/liver weight (AU)	20.0 ± 2.3	20.6 ± 6.8				
Cerebral/ birth weight (AU)	56.3 ± 16	41.6 ± 7.1				
% Brain water content	82.81 ± 0.22	84.35 ± 0.54 *				

Abbreviations: Nx, normoxia; Hx, hypoxia; GD, gestational days; mm, millimeter; g, grams; PI, pulsatility index; AU, arbitraly unit. * Statistical significance, *p* < 0.05.

## Data Availability

The original contributions presented in this study are included in the article and Supplementary Materials. Further inquiries can be directed to the corresponding author.

## Supplementary Materials

The following supporting information can be downloaded at: https://www.mdpi.com/article/10.3390/antiox14060736/s1.

## Abbreviations

The following abbreviations are used in this manuscript:

N	Normoxia
Hx	Hypoxia
GD	Gestational Days
FGR	Fetal Growth Restriction
ROS	Reactive Oxygen Species
NOX	NADPH Oxidase
SOD	Superoxide Dismutase
CAT	Catalase
GPX	Glutathione Peroxidase
H_2_O_2_	Hydrogen Peroxide
GSH	Glutathione (reduced form)
GSSG	Glutathione (oxidized form)
4-HNE	4-Hydroxynonenal
NT	3-Nitrotyrosine
TUNEL	TdT-mediated dUTP Nick-End Labeling
DAPI	4′,6-Diamidino-2-phenylindole
COX2	Cyclooxygenase-2
IL-1β	Interleukin 1 beta
IL-8	Interleukin 8
IL-10	Interleukin 10
TNFα	Tumor Necrosis Factor Alpha
iNOS	Inducible Nitric Oxide Synthase
NF-κB	Nuclear Factor kappa-light-chain-enhancer of activated B cells
BAX	Bcl-2-associated X protein
Bcl-xL	B-cell lymphoma-extra large
Casp-3	Caspase-3
OXPHOS	Oxidative Phosphorylation
ATP	Adenosine Triphosphate
UA	Umbilical Artery
MCA	Middle Cerebral Artery
PI	Pulsatility Index
CPR	Cerebroplacental Ratio
HIE	Hypoxic–Ischemic Encephalopathy
APAF1	Apoptotic Protease Activating Factor 1
ELISA	Enzyme-Linked Immunosorbent Assay
SEM	Standard Error of the Mean
ARRIVE	Animal Research: Reporting of In Vivo Experiments
CICUA	Institutional Committee for the Care and Use of Animals
NIH	National Institutes of Health
