# Supplementary material for: Redox Imbalance Is Associated with Neuronal Apoptosis in the Cortex of Neonates Gestated Under Chronic Hypoxia
Source: Antioxidants (Basel). 2025 Jun 15;14(6):736. doi: 10.3390/antiox14060736 (PMC12189337; doi:10.3390/antiox14060736)
Supplement: Supplementary file 1 [file antioxidants-14-00736-s001.zip › Figure supplemmentary S1.pdf]

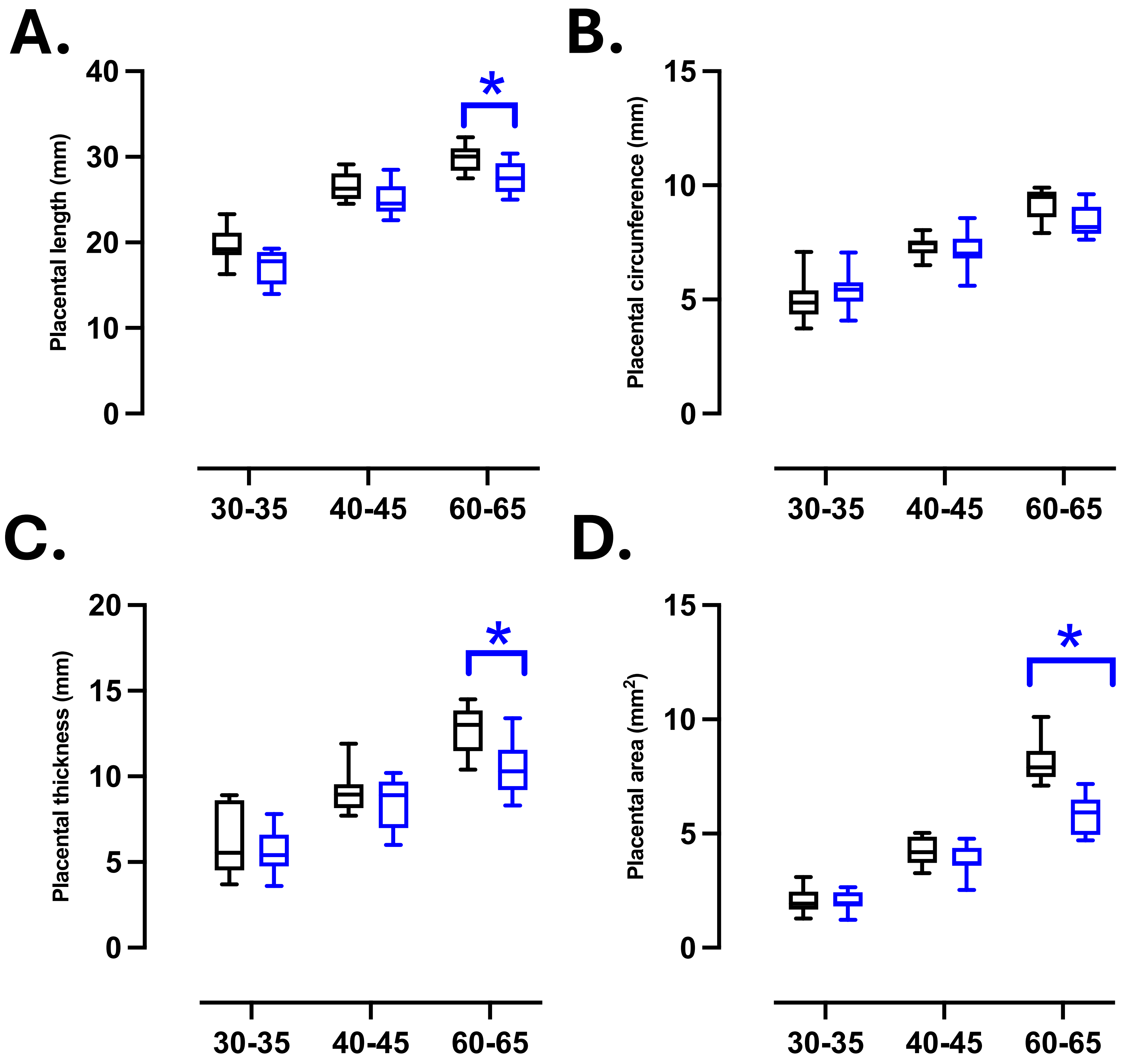

Figure supplemmentary 1. Placental biometry across pregnancy. Guinea pig placental (A) length, (B) circumference, (C) thickness, and (D) área. Groups are *Guinea pig* fetuses gestated in normoxia (Nx, n=10, black circle) or hypobaric hypoxia (Hx, n=10, blue circle). Data are expressed as the mean ± SEM, Multiple unpaired t-tests were used to compare the placental biometry, data were analyzed using a Mann-Whitney t-test. Significant differences ($P \leq 0.05$): * vs. Hx.
